# Cancer Cell Metabolism in Hypoxia: Role of HIF-1 as Key Regulator and Therapeutic Target

**DOI:** 10.3390/ijms22115703

**Published:** 2021-05-27

**Authors:** Vittoria Infantino, Anna Santarsiero, Paolo Convertini, Simona Todisco, Vito Iacobazzi

**Affiliations:** 1 Department of Science, University of Basilicata, Viale dell’Ateneo Lucano 10, 85100 Potenza, Italy; vittoria.infantino@unibas.it (V.I.); anna.santarsiero@unibas.it (A.S.); paolo.convertini@uky.edu (P.C.); 2 Department of Biosciences, Biotechnologies and Biopharmaceutics, University of Bari, Via Orabona 4, 70125 Bari, Italy

**Keywords:** HIF-1, cancer metabolism, hypoxia, TCA cycle, mitochondria

## Abstract

In order to meet the high energy demand, a metabolic reprogramming occurs in cancer cells. Its role is crucial in promoting tumor survival. Among the substrates in demand, oxygen is fundamental for bioenergetics. Nevertheless, tumor microenvironment is frequently characterized by low-oxygen conditions. Hypoxia-inducible factor 1 (HIF-1) is a pivotal modulator of the metabolic reprogramming which takes place in hypoxic cancer cells. In the hub of cellular bioenergetics, mitochondria are key players in regulating cellular energy. Therefore, a close crosstalk between mitochondria and HIF-1 underlies the metabolic and functional changes of cancer cells. Noteworthy, HIF-1 represents a promising target for novel cancer therapeutics. In this review, we summarize the molecular mechanisms underlying the interplay between HIF-1 and energetic metabolism, with a focus on mitochondria, of hypoxic cancer cells.

## 1. Introduction

Cells need appropriate oxygen (O_2_) concentration to allow aerobic respiration, and in turn, to produce ATP, the energetic fuel used in a large number of biological processes. Pathological (cancer, inflammation, diabetes) and non-pathological phenomena (high altitude) may induce hypoxia condition. Hypoxia is a hallmark of tumor microenvironment present in the majority of solid tumors [1] as a consequence of an imbalance between increased oxygen consumption and inadequate oxygen supply due to a rapid cellular proliferation that outgrows the surrounding vasculature [2]. Cancer cells adapt to hypoxia by altering their metabolism through a gene expression reprogramming and proteomic changes that impact various cellular and physiological functions, including energy metabolism, vascularization, invasion and metastasis, genetic instability, cell immortalization, stem cell maintenance, and resistance to chemotherapy [3]. Under hypoxic conditions, cell proliferation is reduced to prevent further increase of O_2_-consuming cells. However, cancer cell mutations and genetic alterations in oncogenes or tumor-suppressor genes together with a highly dynamic metabolic reprogramming allow cell proliferation, despite low-oxygen concentration [4]. Moreover, metabolic rewiring enhances epithelial–mesenchymal transition, invasiveness, and metastatic properties of cancer cells [5].

The metabolic reprogramming, likely through inflammatory mechanisms, also affects distant non-tumor tissues such as adipose tissue and liver and skeletal muscles, inducing an overall energetic dysregulation [6]. This severe state, also called cancer cachexia, leads patients to loss of adipose and muscle mass, weakening, and anorexia, drastically reducing both the quality of life and the effectiveness of anti-cancer treatments [7]. At present, biomarkers addressed to antimetabolic treatments and clarification of the interplay between metabolic rewiring, including hypoxic adaptation, and epithelial–mesenchymal transition are topical fields of investigation [8]. Prevention of metabolic rewiring and resultant adverse events of cancer cells may represent an important approach for the treatment of metastatic cancers.

Among the cellular components, mitochondria are the major consumers of oxygen since they drive cellular bioenergetics, therefore they are severely damaged by decreased oxygen availability. In response to hypoxia, mitochondria adjust their metabolism in different ways, including exchanging or modifying subunits of the respiratory chain, decreasing oxidative phosphorylation (OXPHOS), lowering the tricarboxylic acid (TCA) cycle intermediates, adapting reactive oxygen species (ROS) production, and reducing β-oxidation [9]. Another adaptive response of mitochondria to hypoxic condition involves a reduction of their mass by an autophagy mechanism and inhibition of their biogenesis [10]. Moreover, mitochondrial TCA cycle intermediates participate in modulating the hypoxia-inducible factors (HIF), the key regulators of adaption to hypoxia. 

The transcriptional factor HIF-1 orchestrates the cellular adaptive mechanisms triggered in response to a low-oxygen environment. In fact, most oxygen-regulated genes contain specific responsive elements (hypoxia response elements, HREs) that bind to HIF, leading to an adaptive response to hypoxia and tumor progression and metastasis. Therefore, significant efforts have been made in the last two decades towards understanding the role of HIF family members, in particular HIF-1α, in the adaptive response of cell to hypoxia and in the crosstalk between HIF-1 and mitochondria. This review will focus on the role of HIF-1 in the metabolic reprogramming and in the functional adaption of mitochondria to hypoxia in cancer cells. 

## 2. HIF-1α Structure 

**Figure 1:**
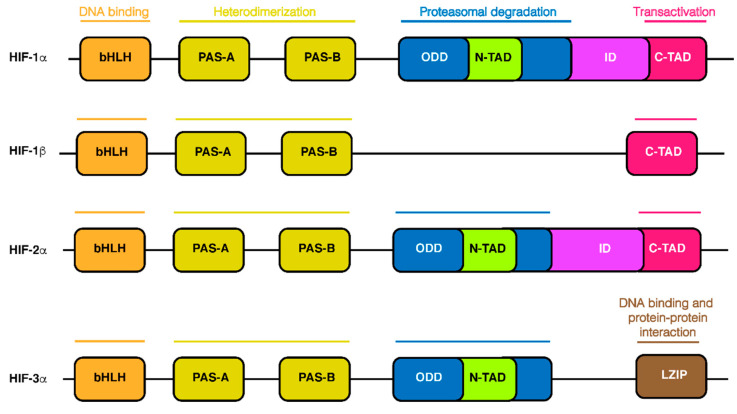
Domain structures of HIF-1α in comparison with other HIF proteins and their potential function. Different functions of various domains are represented at the top. All HIF isoforms have a bHLH motif and two PAS domains (PAS-A and PAS-B) for the heterodimerization. HIF-1β does not contain the oxygen-dependent degradation (ODD) domain, that mediates proteasomal degradation, N-TAD, and the inhibitory domain (ID). Isoform HIF-3α, without the C-TAD which mediates transcriptional activation and ID, shows a LZIP involved in DNA binding and protein–protein interaction.

Three members of the HIF family have been identified. They all consist in a heterodimeric structure composed by an O_2_-sensitive α subunit (HIF-1α, HIF-2α, and HIF-3α) and an O_2_-insensitive β subunit (HIF-1β), also known as aryl hydrocarbon receptor nuclear translocator (ARNT) [11]. Among them, HIF-1α is the most well-characterized isoform thanks to the structural and functional studies performed on mammals. HIF-1α consists of 826 amino acids and HIF-1β of 789 amino acids. Both subunits belong to the basic helix-loop-helix/Per-ARNT-Sim (bHLH-PAS) family of transcription factors. Two regions of amino sequences (PAS-A and PAS-B) and HLH domain, located in the first half of subunits, are responsible for heterodimerization between HIF-1α and HIF-1β (Figure 1). The basic regions upstream of the N-terminal of the HLH domain of both HIF-1α and HIF-1β subunits mediate the binding to the HRE of target gene promoters (Figure 1) [12]. Differences in terms of structure and function are present in the second half of subunits. Two transactivation domains (TADs), N-TAD (N-terminal TAD) and C-TAD (C-terminal TAD), are present in HIF-1α subunit. Both domains are rich in acidic and hydrophobic amino acids. They are separated by a region known as the inhibitory domain (ID) (Figure 1), responsible for suppression of transcriptional activity under normoxic conditions [13]. The C-TAD regulates transactivation of target genes (Figure 1) through the recruitment of coactivators CBP and p300 in both subunits [14]. Upstream of the N-TAD region, HIF-1α contains the oxygen-dependent degradation (ODD) domain that mediates the hydroxylation of two proline residues and the acetylation of a lysine, followed by proteasomal degradation (Figure 1). HIF-1β subunit lacks the ODD and the N-TAD domains but contains C-TAD alone (Figure 1). This structural difference reflects the function of subunits.

In fact, HIF-1α is responsible for transcriptional activity since it contains the transactivation domains, whereas HIF-1β is only the dimerization partner and is not needed for the induction [15]. The oxygen cellular tension regulates the stability and functionality of HIF-1α. While HIF-1β is constitutively stable and active under both aerobic and hypoxic conditions, HIF-1α subunit is rapidly degraded in normoxic conditions by the ubiquitin-proteasome system [16]. Indeed, detection of protein expression in different human tissues can evidence low levels of HIF-1α protein in normoxic cells (due to proteasomal degradation), even when HIF-1α is overexpressed, but a high induction in hypoxic cells. Conversely, HIF-1β levels were stable, regardless of pO_2_ [17]. 

The second member of the HIF family, HIF-2α shares some structural similarities with HIF-1α but has a different pattern of expression and functional properties. Like HIF-1α it shows the same ability to heterodimerize with HIF-1β [18]. Unlike HIF-1α, which is ubiquitous, HIF-2α shows an abundant expression in embryonic cells and adult vascular endothelial cells, lungs, placenta, and heart [19,20,21]. Differences have also been found in their transcriptional targets. As reported in the next sections, HIF-1α is involved in different metabolic pathways, while HIF-2α is more effective on erythropoietin (EPO) gene and on Fe metabolism. Both regulate other genes such as VEGF and GLUT-1 [22,23]. Interestingly, primary renal cell carcinoma revealed a correlation between HIF-2α (but not HIF-1α) and generation of lipid droplets (see Section 3.3).

The third member, HIF-3α, shows 57% and 53% amino acids sequence identity in the bHLH-PAS domain with HIF-1α and HIF-1β respectively, and 61% identity in the ODD domain with HIF-1α [24]. It contains N-TAD domain, but lacks the C-TAD domain (Figure 1). Of note, a leucine zipper (LZIP) domain, which is responsible for interaction between proteins, was found in a HIF-3α variant [25,26]. Additionally, this member can heterodimerize with HIF-1β. Experimental evidence shows that, under hypoxia, it is expressed in adult mice thymus, lung, brain, heart, and kidney [27]. Multiple HIF-3α splice variants have been found, but their specific functional activity is still unknown. Interestingly, they all are induced by hypoxia via the involvement of HIF-1α, but not HIF-2α [28]. 

**Figure 2:**
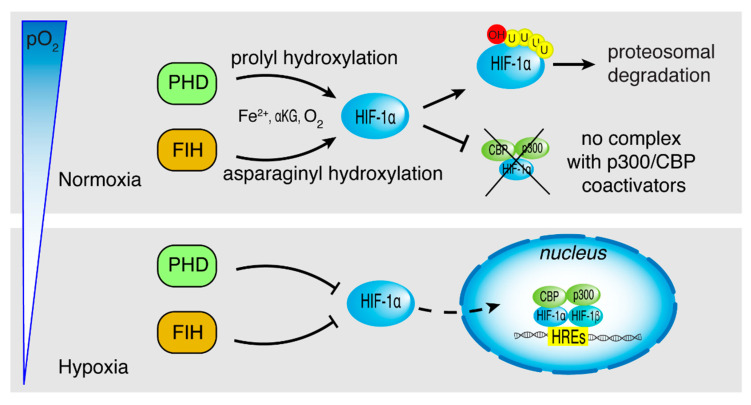
Oxygen-dependent regulation of HIF-1α. Under normoxia (upper panel), HIF-1α proline hydroxylation promotes proteasomal degradation, while HIF-1α asparagine hydroxylation blocks CBP/p300 binding, thus impairing HIF-1α function. Both prolyl (PHD) and asparaginyl (FIH) hydroxylases require Fe(II), O_2_, and α-ketoglutarate (αKG). Under hypoxia (lower panel), HIF-1α is not hydroxylated, and its nuclear translocation allows the dimerization with HIF-1β, the combining with the coactivators CBP/p300, and thereby, the binding to hypoxia response elements (HREs), increasing the transcription of target genes.

The mechanism by which HIF-1α is degraded in normoxia involves the post-transcriptional hydroxylation of P402 and P564 within the ODD domain by prolyl hydroxylase domain protein 2 (PHD2). The enzyme requires molecular oxygen, α-ketoglutarate (αKG), and Fe(II) and generates succinate and CO_2_ as by-products (Figure 2) [29]. In the oxygenated form, HIF-1 recognizes the von Hippel-Lindau tumor suppressor protein, which binds ubiquitin-conjugated E2 component to assemble the protein complex for the degradation by the ubiquitin-proteasomal pathway. Another HIF-1α hydroxylation reaction occurs in well-oxygenated cells: the hydroxylase called factor inhibiting HIF-1 (FIH-1), a Fe(II)- and αKG-dependent dioxygenase, hydroxylates N803 with consequent disturbance of the interaction between HIF-1α and transcription coactivators CBP/p300 (Figure 2) [14,30]. Under hypoxic conditions, PHD2 and FIH-1 lose their activity, giving rise to stabilization, nuclear translocation of HIF-1α, and activation of HIF target genes (Figure 2) [31]. As a consequence of reduced pO_2_, mitochondria increase the production of ROS which oxide Fe(II) present in the active sites of dioxygenases, causing their inactivation [32]. It is also known that HIF-1α can be stabilized by ROS generated by other mechanisms such as the NADPH oxidase system, in particular the NOX family of oxidase [33]. 

## 3. Metabolic Reprogramming in Hypoxia Induced by HIF-1

Cancer cell metabolism is reprogrammed in hypoxia, thus taking advantage from these adaptions to fuel survival, proliferation, and ensure tumor progression. Glucose metabolism is particularly affected. Recent advances also highlighted the active involvement of HIF-1 in the regulation of the metabolism of two amino acids, glutamine and serine, together with one-carbon cycle and the fatty acid metabolism in hypoxic cancer cells.

### 3.1. Glucose Metabolism

Cancer cells use more glucose than normal cells. In hypoxia, different genes involved in glycolysis are under HIF-1 transcriptional control, such as glucose transporters (GLUT1 and GLUT3), glycolytic enzymes (i.e., hexokinase 1 and 2, enolase 1, phosphoglycerate kinase 1, pyruvate kinase M2), and lactate dehydrogenase (LDHA) [34,35]. Among them, the pyruvate kinase M2 gene (*PKM2*) plays different roles since it encodes two isoforms, PKM1 and PKM2, by an alternative splicing mechanism. In cancer cells, expression of PKM2 is induced, promoting cell proliferation [36]. Once activated, PKM2 triggers different biological pathways as a transcriptional activator for octamer-binding transcription factor 4, HIF-1α, HIF-2α, and β-catenin [37,38]. PKM2 enzyme acts as a coactivator of HIF-1α itself to stimulate chromatin binding, coactivator recruitment, and transcriptional activation [39], leading to a metabolic reprogramming of cancer cells and favoring other processes of cancer progression, such as angiogenesis. Furthermore, PKM2 can also phosphorylate the transcriptional factor STAT3 [40]. However, PKM2 mainly acts as pyruvate kinase in glycolysis, although it is differently regulated than its isoform PKM1. In fact, its activity is regulated in an allosteric way by phosphopeptides [41], metabolites [42], and other post-translational modifications such as phosphorylation, acetylation, and oxidation [43,44,45]. Generation of different glycolytic intermediates can be used for nucleotide and lipid synthesis [46]. In normoxia, pyruvate is converted to acetyl-CoA by pyruvate dehydrogenase complex (PDH) for entry into TCA cycle. In hypoxia, PDH is inactivated by phosphorylation at its catalytic domain by pyruvate dehydrogenase kinase 1 (PDK-1) induced by HIF-1, giving rise to a reduced delivery of NADH and FADH_2_ to electron transport chain (ETC) to generate ATP [3]. Induction of glycolysis together with inhibition of PDH favors production of lactate, which stabilizes HIF-1, a metabolic process very active in tumor cells characterized by a shift from OXPHOS to anaerobic glycolysis [47]. 

In the context of glucose metabolism reprogramming in hypoxia, the pentose phosphate pathway activity also decreases due to a reduced expression of glucose-6-phosphate dehydrogenase. As a consequence, synthesis of nucleotides and cell proliferation are affected. Contextually, phosphoglycerate dehydrogenase is induced, resulting in a diversion of glucose towards serine synthesis, as described later. 

Finally, under hypoxia, HIF-1 also stimulates glucose conversion into glycogen, thus ensuring energy storage to survive prolonged stress. Depending on the metabolic demands of specific cancer cells, glycogen metabolism is subjected to a dynamic process of synthesis and degradation in hypoxic conditions of cancer cells. Different enzymes involved in glycogen synthesis are induced, including glycogen synthase 1, glycogen branching enzyme, UDP-phosphorylase, and phosphoglucomutase 1 [34]. The glycogen phosphorylase enzyme involved in the degradation of glycogen is highly expressed in cancer cells. This enzyme has been tested in pancreatic cancer and U87 glioma as a therapeutic target by its inhibitor C-320626 [48,49]. Taken together, these studies indicate a primary role for HIF-1 in modulating the dynamic glucose metabolism of hypoxic cancer cells.

### 3.2. Lactate and Acidification

As a result of the hypoxia-related metabolic changes, the lactate concentration ranges from 1.5 to 3 mM in normal cells to 10–30 mM in cancer cells, resulting in intracellular acidification [50,51]. To avoid this effect, an efflux of lactate and H^+^ to the extracellular space occurs through proton-like monocarboxylate transporters (MCTs) [52,53]. Lactate export is also required to support high glycolytic rates, as increased cytosolic lactate levels inhibit phosphofructokinase 1 (PFK1) catalyzing one of the key rate-limiting steps of glycolysis [54]. Lactate efflux together with high activity of the carbonic anhydrase IX—which reversibly hydrates carbon dioxide to bicarbonate ions and H^+^—induces an acidification of the extracellular pH (6.0–6.5) in tumor microenvironment [55]. Acidic extracellular pH represents a hallmark of cancer. These events make the environment unfavorable for cell growth and help tumor progression since cancer cells have developed more adaptation than normal cells to these conditions. Moreover, extracellular lactate-driven acidification promotes immune evasion by inhibiting activation and proliferation of CD4, CD8, NK, NKT, and dendritic cells, and by leading to apoptosis of CD8 lymphocytes and NK cells [56]. Thus, hypoxia-induced tumor microenvironment acidification lowers antitumor immunity. Lactate itself could be a respiratory substrate in cancer cells [57]. Lactate and H^+^ are exported outside cells by the monocarboxylate transporter, carbonic anhydrase 9 and Na^+^/H^+^ exchanger isoform 1 [58]. Extracellular lactate can be used as an anaplerotic source of substrates for TCA cycle, triggered in cancer cells. In particular, evidence from Faubert et al. shows that lactate is the preferred anaplerotic substrate in human non-small-cell lung cancers since cancer cells incorporate more lactate-derived carbons into TCA cycle than those from glycolysis [59]. Moreover, excreted lactate contributes to regulate pH homeostasis inside the cell and acidification of the extracellular space. Lactate and/or protons in the microenvironment regulate the function of immune cells and promote invasion and metastasis [60]. Since T cells also depend on glycolysis, cancer cells and T cells compete for glucose uptake. However, tumor cells take up most of the glucose and thereby create a state of glucose deprivation for T cells, leading to T cell anergy or death [61].

### 3.3. Lipid Metabolism

Cancer cells have an increased lipid content [62]. In hypoxic conditions, HIF-1 induces lipid droplet accumulation and uptake, and reprograms β-oxidation and ex novo biosynthesis of fatty acids. Regarding the uptake, FABP3 and FABP7, the fatty acid binding proteins, and ADRP, the factor required for droplet formation, are induced by HIF-1. This condition protects against ROS and contributes to cell growth and survival after hypoxia regeneration [63]. 

HIF-1 suppresses the mitochondrial fatty acid oxidation through inhibition of medium-chain Acyl-CoA dehydrogenase (MCAD) and long-chain Acyl-CoA dehydrogenase (LCAD), resulting in reduction of ROS and suppression of the PTEN pathway, thus promoting tumor cell proliferation [64]. Inhibition of fatty acid oxidation contributes to maintain redox homeostasis in hypoxia. HIF-1α suppresses the expression of c-Myc, a transcriptional coactivator of PGC-1β, required for MCAD and LCAD. Interestingly, carnitine palmitoyltransferase 1A, the rate-limiting component of mitochondrial fatty acid transport, is repressed by HIF-1 and HIF-2, resulting in reduction of transport into mitochondria and forcing fatty acids to lipid droplets formation [65].

Lipid biosynthesis becomes critical in high proliferating cancer cells to support organelle membrane production and modulation of their fluidity, to supply triglyceride formation for energy storage, and to produce signaling molecules such as sphingosine 1-phospahte and lysophosphatidic acid involved in the cancer cell migration, inflammation, and survival [66]. In hypoxia, the precursor of fatty acid biosynthesis, acetyl-CoA, comes from alternative sources than the normal glucose pathway. In addition to glutamine or some amino acids, acetyl-CoA derives from acetate through the cytosolic acetyl-CoA synthetase. Acetyl-CoA not only serves as a substrate for fatty acid biosynthesis, but it also acetylates HIF-2 by its coactivator CBP, leading to increased cancer cell motility and invasion [67].

HIF-1 also promotes the expression of fatty acid synthase to trigger fatty acid synthesis and stearoyl-CoA desaturase (SCD) to stimulate the unsaturated fatty acid generation [35]. In hypoxic conditions, the ratio between saturated/unsaturated fatty acids is altered because the oxygen-dependent SCD enzyme is inhibited [68], thus affecting plasma and organelle membrane integrity and cell function. This imbalance is compensated by the induction of expression of SCD, as observed in some type of tumors, such as prostate, breast, liver, and kidney cancers [69]. Maintaining the saturated/unsaturated fatty acid ratio is important since induction of saturated fatty acid biosynthesis is harmful if not compensated. In fact, toxicity derived from saturated fatty acid induction is alleviated by exogenous unsaturated fatty acid intake in cancer cells [68]. Moreover, ATP citrate lyase (ACLY) is upregulated in hypoxic tumor cells and seems to be a target gene of HIF-1α [70]. Indeed, as an enzyme at the interface between glucose and lipid metabolism, ACLY is essential not only for fatty acid biosynthesis but also for acetyl-CoA production [71]. ACLY is also a component of the citrate/pyruvate shuttle important for NADH oxidation and NADPH production for lipid metabolism, redox homeostasis, and molecular biosynthesis [72].

The induced synthesis of fatty acids leads to an increased production of neutral triacylglycerols (TAGs), stored as lipid droplets (LDs) to be used as energy depots. Two main enzymes, AGPAT2 (acylglycerol-3-phosphate acyltransferase 2) and lipin-1 (a phosphatidic acid phosphatase) are induced by HIF-1 mediating LD accumulation [73,74]. Both enzymes are necessary not only for LD accumulation and viability, but in the onset of chemoresistance under hypoxia [75]. The products of AGPAT2 catalytic activity, lysophosphatidic acid (LPA) and phosphatidic acid (PA), can also be used for new membrane formation [76]. Hypoxic conditions also induce expression of other constituents of LD membranes. Transcriptional profiling of primary renal cell carcinoma revealed a correlation between HIF-2α (but not HIF-1α) and high expression of the lipid droplets coat protein perilipin 2 (PLIN2). The HIF-2α-dependent PLIN2 expression promoted triglycerides and cholesterol storage in lipid droplets needed for maintaining integrity of the endoplasmic reticulum and its homeostasis, particularly under conditions of nutrient and oxygen limitation, thereby promoting tumor cell survival [77]. In addition, HIF-1 increases lipid accumulation in both cancer and normal cells by induction of HIG2/HILPDA (hypoxia-inducible protein 2/hypoxia-inducible lipid droplet-associated) [78,79]. As a consequence of HIG2 upregulation, the adipose triglyceride lipase (ATGL) is inhibited, resulting in impairment of intracellular lipolysis in various cancer cells [80].

Another aspect of lipid metabolism concerns the lipid droplets’ composition. Interestingly, Ackerman et al. found that, in hypoxia, the unsaturated fatty acid oleate is preferentially released from lipid droplets triglycerides into the phospholipids pool to counter the build-up of saturated lipids [81]. Finally, it should be mentioned that different lipid signaling molecules, such as sphingosine kinase 1, stimulate HIF-1 activity [82].

### 3.4. Amino Acids’ Metabolism

Metabolism of two non-essential amino acids, glutamine and serine, is particularly affected by hypoxia in cancer cells. These amino acids are more than a simple substrate for protein synthesis since their metabolism is crucial for proliferating cells. 

Glutamine is one of the primary substrates in cancer cells. It can be utilized to supply TCA cycle, to support biosynthesis, energetics, and cellular homeostasis as a source of carbon and nitrogen. The decreased entry of pyruvate into TCA cycle triggers a compensatory anaplerosis consisting in the increase of glutamine uptake through induction of glutamine transporters SLC1A5 and SNAT2/SLC38A2 [83,84]. Inside cells, glutamine is converted into glutamate by glutaminase and then into αKG by glutamate dehydrogenase or transaminase. αKG can be converted into succinate for entry into TCA cycle or subjected to reductive carboxylation by isocitrate dehydrogenase (IDH), giving rise to isocitrate and citrate [85]. A shift from oxidation to reductive carboxylation occurs in hypoxic conditions by a mechanism involving HIF-1. In hypoxic glioblastoma cells, most of the citrate comes from glutamine through reductive carboxylation. The same cell line is unable to proliferate in citrate starvation or *IDH2-*silencing [86]. 

Oxidation of αKG also depends on HIF-1 in hypoxic cancer cells. In fact, HIF-1 activation promotes SIAH2-targeted ubiquitination and proteolysis of the 48 kDa splice variant of the E1 subunit of the α-ketoglutarate dehydrogenase complex. Under induction of HIF-1, an increase of the glutamine utilization in P493 cells (model of Burkitt lymphoma) has been observed [87]. Furthermore, in clear cell renal cell carcinoma, the presence of inactivating mutations of the von Hippel-Lindau tumor suppressor gene stabilize HIF proteins, which accumulate to supraphysiologic levels and activate the transcription of genes such as vascular endothelial growth factor and platelet-derived growth factor. All these events contribute substantially to the tumor physiology and have been assessed indirectly as a prognostic factor [88]. In these cancer cells, glutamine is used to generate aspartate needed for de novo pyrimidine synthesis via reductive carboxylation [89]. Glutamine-derived glutamate may be utilized for other biological processes, such as glutathione synthesis, transamination reaction, precursor of other amino acids’ biosynthesis, and to drive the tumor growth [90,91]. 

Serine plays a central role in cancer cells’ growth. In fact, its depletion inhibits the growth of some cancer cells in vitro and in vivo [92]. It exerts a multifunctional role: the synthesis of other amino acids (glycine and cysteine), production of phospholipids (phosphatidylserine), and donor of one-carbon units in the folate pathway. In hypoxia, serine synthesis from glucose is induced by three enzymes: phosphoglycerate dehydrogenase (PHGDH), phosphoserine amino transferase, and phosphoserine phosphatase. PHGDH is overexpressed in some types of cancer, such as non-small cell lung, cervical, colorectal, and breast cancers [93,94,95]. However, Samanta et al. showed that some breast cancer cell lines have no PHGDH overexpression, which can be amplified only in an HIF-dependent manner under hypoxic conditions [96]. Furthermore, HIF-1 activates the transcription of SLC7A11, encoding the cysteine transporter, and GCLM, encoding the regulatory subunit of the glutamate-cysteine ligase, to increase glutathione synthesis.

One-carbon cycle is strictly related to the serine metabolism [97]. It is involved in the NADPH generation and redox regulation. Specifically, NADPH is necessary for the conversion of glutathione from oxidized (GSSG) to reduced (GSH) form and to protect against increased mitochondrial ROS generated by ETC [98]. In hypoxia, breast cancer cells produce NADPH to maintain the reduced form of glutathione and support the cell survival. PHGDH knockout reduces production of NADPH and increases ROS amount, resulting in increase of apoptosis [99]. Three mitochondrial enzymes of the folate cycle, hydoxymethyltrasferase 2 (SHMT2), methylene tetrahydrofolate dehydrogenase, and methylene tetrahydrofolate dehydrogenase 1-like, are induced by HIF-1 in hypoxic conditions. Mitochondrial degradation of serine to CO_2_ and NH_4_^+^ by SHMT2 increases a net production of NADPH favoring antioxidant defenses in hypoxic cells. In fact, knockdown of SHMT2 in MYC-dependent cells or in vivo suppression of SHMT2, in addition to reduction of cellular NADPH, increases ROS production, triggers hypoxia-induced cell death, and impairs tumor growth [100].

## 4. Impact of Hypoxia on Mitochondrial Function

Mitochondria play a critical role in cancer cells. Thus, in hypoxic conditions, cancer cells rewire important mitochondrial functions and biogenesis. Different mitochondrial processes, such as oxidative phosphorylation, TCA cycle, ROS generation, as well as mitochondrial dynamics (i.e., mitophagy), are significantly affected by decreased oxygen availability. In this section, we summarize mitochondrial metabolic and molecular changes and the role of HIF-1 as a master regulator of mitochondrial redox homeostasis by decreasing oxidant production and increasing antioxidant capacity in hypoxia

### 4.1. TCA Cycle

**Figure 3:**
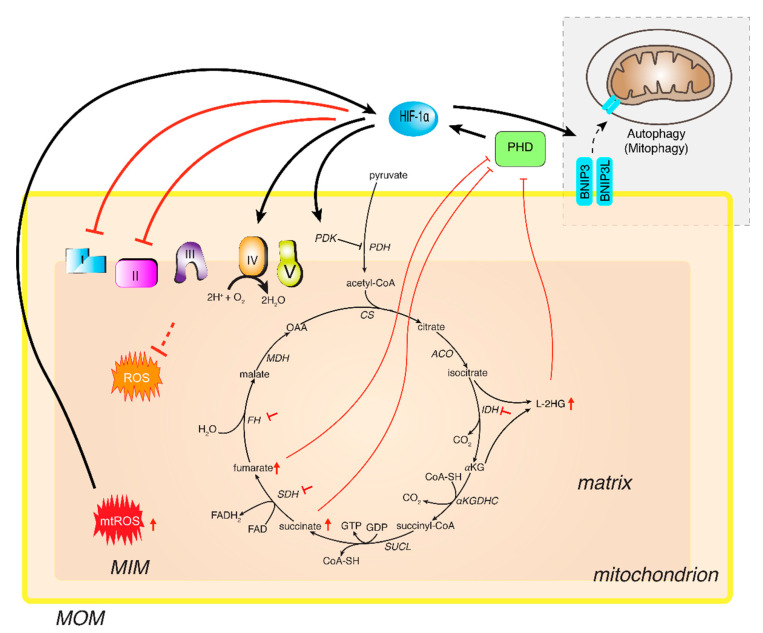
HIF-1 and mitochondrial function. Under hypoxic conditions, cancer cells activate HIF-1, and its interplay with mitochondria is critical for cell survival. HIF-1α impairs pyruvate conversion to acetyl-CoA by activating pyruvate dehydrogenase kinase (PDK). Two proteins belonging to the BCL2 family of mitochondrial proteins (BNIP3 and BNIP3L) trigger mitophagy following activation by HIF-1. Dysregulation and/or mutations of genes encoding succinate dehydrogenase (SDH), fumarate hydratase (FH), and isocitrate dehydrogenase (IDH) TCA cycle enzymes lead to accumulation of succinate, fumarate, and L-2-hydroxyglutarate (L-2HG), which decrease the activity of PHD, resulting in a stabilization of HIF-1α subunit. HIF-1 induces a reduction of the mitochondrial Complex I, Complex II, and improves Complex IV activity in transferring electrons to oxygen in hypoxic condition, thereby lowering ROS production and preserving the membrane potential. Mitochondrial ROS (mtROS) activate HIF-1. Abbreviations: CS: citrate synthase; ACO: aconitase; IDH: isocitrate dehydrogenase; αKGDHC: αKG dehydrogenase complex; SUCL: Succinyl-CoA ligase; FH: fumarate hydratase; MDH: malate dehydrogenase; PDH: pyruvate dehydrogenase; PHD: oxygen-dependent prolyl hydroxylase; L-2HG: L-2-hydroxyglutarate; αKG: α-ketoglutarate; Ac-CoA: Acetyl-Coenzyme A.

It is known that TCA cycle plays a dual role as a producer of NADH and FADH_2_, which fuel the mitochondrial electron transport chain to generate ATP, and as a supplier of metabolic intermediates required for anabolic reactions. Highly proliferating cancer cells require a continuous supply of precursors for the synthesis of lipids, proteins, and nucleic acids in hypoxic conditions in which a reprogramming of TCA cycle is triggered. Inactivation of PDH at its catalytic site by the PDK-1 phosphorylation significantly reduces the conversion of pyruvate to acetyl-CoA, thus reducing the TCA cycle flux, which limits the availability of reducing equivalents for the ETC. Under hypoxic conditions, reduction of TCA cycle activity lowers the levels of aspartate that is generated form the TCA cycle metabolite oxaloacetate. Aspartate is needed for nucleotide synthesis and cell proliferation; thus, its reduction impairs cell proliferation in vitro and cancer cells’ growth in a mouse model [101,102]. Mutations in the TCA cycle components, such as succinate dehydrogenase (SDH), fumarate hydratase, and isocitrate dehydrogenase (IDH1 and IDH2), lead to accumulation of succinate, fumarate, and L-2-hydroxyglutarate (L-2HG), respectively [103]. These metabolites decrease the activity of PHDs (Figure 3), resulting in a stabilization of HIF-1α subunit, which accelerates multiple steps of metastasis [104]. On the contrary, another TCA cycle intermediate, αKG, acts as a co-substrate of PHD. However, αKG accumulation promotes the production of L-2HG oncometabolite via LDHA and malate dehydrogenase activity in the cytosol (MDH1), and in the mitochondria (MDH2) [105]. The L-2HG enantiomeric form in turn inhibits PHDs. Therefore, dysregulations of TCA cycle enzymes and/or metabolites regulate PHD function, and consequently HIF-1α stability. These studies concerning the dysregulation of TCA represent a molecular strategy by which hypoxic cells decrease oxidative metabolism. 

### 4.2. Electron Transport Chain

The electron transport chain contains different redox centers that transport electrons to oxygen, producing two molecules of water. Complex IV, the last component of the electron transport chain, shows a high affinity for oxygen (K_m_ close to 0.1% O_2_) to ensure production of ATP, also during hypoxia. However, prolonged hypoxic state may lead to a reduction of ETC function. Unexpectedly, reduction of ATP activity does not depend on the oxygen deficiency, but it is mainly based on a HIF-1-dependent mechanism. Complex IV, also known as cytochrome oxidase (COX), consists in 13 subunits (10 nuclear DNA-encoded and 3 mitochondrial DNA-encoded). In hypoxia, HIF-1 induces the expression of the nuclear-encoded subunit COX4I2 and the mitochondrial LON protease. COX4I2 replaces COX4I1 in complex IV assembly and then COX4I1 is targeted by LON for proteasomal degradation [3]. Incorporation of COX4I2 improves the complex activity in transferring electrons to oxygen in hypoxic condition, resulting in reduction of ROS production, maintenance of ATP production, and preserving the integrity of Complex IV (Figure 3). Efficiency of Complex IV is ensured by the action of HIF-1, a positive regulator of COX, which causes structural changes around heme *a*, the active center driving the proton pump [106]. 

HIF-1 increases expression of the mitochondrial NDUFA4L2 encoding NADH dehydrogenase (ubiquinone) 1/subcomplex subunit 4-like 2, which suppresses Complex I activity [107]. Reducing the mitochondrial Complex I activity via NDUFA4L2 is an essential process in the mitochondrial reprogramming induced by HIF-1 that leads to a reduction of intracellular ROS production and preservation of membrane potential (Figure 3). Interestingly, NDUFA4L2 knockdown suppresses tumor growth and metastasis in vivo [108]. 

miRNA-210 (miR-210) is another established target of HIF-1 in the adaption of respiration in hypoxic conditions aimed to decrease oxidative metabolism. This small RNA inhibits expression of iron–sulfur cluster assembly proteins (ISCU1 and ISCU2) required for Complex I assembly [109]. Decrease of ISCU induces ROS formation under hypoxia [110]. Although NDUFA4L2 and ISCU1/2 are both HIF-1-regulated proteins, they play an opposite role in the production of ROS in hypoxia. While NDUFA4L2 induction increases ROS, reduction of ISCU decreases ROS production. Furthermore, hypoxia also affects the Complex I conformation change from an active (ROS-producing) to silent (ROS not producing) form, resulting in a decrease of ROS. Although the mechanism is not fully understood [111], it is likely that subunits, such as NDUFA9, ND1, and ND3, are involved in this process [112]. Furthermore, HIF-1-induced miR-210 also targets succinate dehydrogenase, a membrane-bound and heme *b*-containing subunit of Complex II of the ETC. Expression of SDH decreases in A549 cells transfected with miR-210, resulting in a lower activity of Complex II [113]. In addition to Complex I and II, Complex IV subunits, COX10 and NDUFA4, have been identified as miR-210 targets [114,115]. Therefore, HIF-1 may regulate ETC directly or through miR-210 in hypoxic cancer cells. These data concerning the replacement of distinct proteins, altering the function of the complexes of ETC, can be considered a fast and flexible system to adapt to hypoxic conditions without building an entire new complex. 

### 4.3. Mitochondrial Biogenesis and Autophagy

HIF-1 activates BCL2 interacting protein 3 (BNIP3) and BNIP3-like (BNIP3L), two mitochondria-associated proteins belonging to the BCL2 family, which trigger mitochondrial selective autophagy (Figure 3). Mitophagy [116] is an adaptive mechanism to face the reduced oxidative metabolism in order to maintain oxygen homeostasis and contribute to metabolic reprogramming in hypoxic conditions of cancer cells [117,118]. In different cancers, autophagy is also repressed through glutamine by suppressing GCN2 (amino acid-sensing kinase) and stimulating mTOR [119]. In addition to induction of autophagy, HIF also suppresses mitochondrial biogenesis by transactivation of MXI1, a transcriptional repressor that negatively regulates MYC function. All together, these mechanisms play a protective role by decreasing ROS production in mitochondria. Thus, they can be considered as part of a general mechanism of cell survival that is controlled by HIF-1. 

### 4.4. ROS

Though ROS can be produced by NADPH oxidase and non-enzymatic mechanisms, mitochondria are the powerful source of ROS (mtROS) by ETC, especially superoxide anion (O_2_^•−^) produced at the level of Complex I and Complex III, and ROS-detoxifying enzymes. Hypoxia leads to mtROS production, alerting cells to a shortage of oxygen. In turn, mtROS activate HIF transcription factors and induce the expression of HIF target genes, including those involved in metabolism and angiogenesis (Figure 3). A steady-state level of ROS is needed in cancer cells to allow cell proliferation and HIF activation. This redox balance is also aimed to avoid accumulation of ROS that would incur cell death or senescence. Thus, mtROS levels are tightly regulated in cancer cells. Serine catabolism through one-carbon metabolism maintains this mitochondrial redox balance during hypoxia [100]. Interestingly, cancer cells can protect mitochondria under hypoxia by an increased expression of antioxidant proteins [120]. Thus, cytoplasmatic and intermembrane space superoxide dismutase 1 (SOD1) and mitochondrial matrix SOD2 are upregulated in hypoxic conditions, enhancing superoxide detoxification and producing H_2_O_2_. These findings highlight that mitochondrial-derived ROS can be considered not only harmful substances but important signaling molecules, and their level has to be strictly controlled to avoid cell death.

## 5. Targeting HIF-1 in Cancer Therapy

Since the HIF system and related hypoxia are crucial events in cancer cells, they have been proposed as possible therapeutic targets in cancers. In recent years, different strategies and drugs have been tested and proposed as therapeutic tools to impair the HIF activity and related pathways, but only few of them are in clinical trials due to tolerance limitations, lack of hypoxic selectivity, or specificity on HIF isoforms. The basic approach consists in the inhibition of different steps of the HIF activation pathway: transcription, translation, stability, transport into nucleus, heterodimerization, binding to DNA and transcriptional activity, and HIF target genes. HIF-1α inhibitors have been more extensively investigated than those of HIF-2α. Below, we briefly report the main aspects of the inhibition strategy and examples of chemical agents used. For a more detailed discussion about drugs, targets, and mechanisms, more specific reviews should be consulted [121,122,123]. 

Considering the whole HIF pathway from the beginning, the first target of inhibition can be transcription and translation processes. Transcription can be inhibited by different kinds of compounds, such as aminoflavone [124], GL331 [125], and anthracyclines [126]. Other compounds belonging to cardiac glycosides [127], steroids [128], topoisomerase inhibitors [129], and microtubule binding agents [130] show the inhibitory effect of translation rate of HIF mRNA by different mechanisms. However, these compounds lack specificity on isoforms. Interestingly, YC-1 shows a specificity to HIF-1, but not to HIF-2, in macrophages [131]. Inhibition of HIF-1α translation by digoxigenin increases sensitivity of pancreatic cancer cells to gemcitabine [132].

A second target for inhibition can be HIF stability. Some inhibitors weaken stability of HIF through the induction of its degradation. Panobinostat, a histone deacetylase inhibitor, disrupts the HSP90/HDAC6 complex, which normally interacts with HIF-1α to prevent its degradation, thus blocking of complex formation induces HIF-1α degradation more rapidly [133]. Similarly, another histone deacetylase inhibitor, MPT0G157, decreases HIF-1α in colorectal cancer [134]. Other HDAC inhibitors, such as Vorinostat, inhibit hypoxia machinery through downregulation of HIF-1α and VEGF. Currently, Vorinostat is used to treat cutaneous T cell lymphoma [135]. Promising results are expected from other approved HDAC inhibitors: romidepsin [136], belinostat [137], panobinostat [138], and chidamide [139]. Furthermore, rapamycin induces HIF-1 degradation and enhances downregulation of survivin, an inhibitor of apoptosis expressed in countless malignancies. Antioxidant agents, such as N-acetyl cysteine, impair HIF-α subunit [140].

A crucial step for HIF activation is the heterodimerization process. Chemical agents used in this strategy target the PAS domains of HIF-1α and HIF-2α, resulting in inhibition of heterodimerization. Interestingly, exploiting structural differences in PAS-B domain between HIF-1α and HIF-2α, compounds PT2399 and PT2385 have been found to specifically inhibit HIF-2α activity and showed anticancer properties in both cellular and animal renal carcinoma (ccRCC). Patients with ccRCC and recurrent glioblastoma treated with these inhibitors exhibited very promising results [141,142], thus they are under investigation in phase II clinical trials. Encouraging results are expected from the second-generation HIF-2α inhibitors, PT2977 and 0 × 3 [143]. Acriflavine, belonging to a different class of compounds, can also block binding of PAS-B domain of HIF-1α and HIF-2α to HIF-1β [31]. HIF, as a transcriptional factor, exerts it activity into the nucleus. Thus, impairment of HIF nuclear localization can be another strategy to target. Inhibition of ERK1/2 pathway by natural products (e.g., Kaempferol) and chemicals (PD98059 and U0126) affects the phosphorylation of both HIF-α proteins and triggers CRM1-dependent nuclear export of HIF-α. Consequently, transcriptional activity of HIF is hampered [144,145]. Other targets include the DNA binding and transcriptional activity. Chemical agents, such as echinomycin and doxorubicin, impair binding of HIF to chromatin, resulting in blocking of its transcriptional activity [146,147]. Alternatively, agents (e.g., chetomin) affect the ability of HIF to form complex with transcriptional coactivators CBP/p300 [148]. Inhibition of HIF transcriptional coactivators, such as CBP and p300, can also be an attractive strategy to inhibit the HIF pathway. Two domains, the histone acetyltransferase [149] and the bromodomain (BRD), are present in these coactivators. Thus, drugs addressing these domains may affect interaction of coactivator with HIF. Two BRD inhibitors of p300 are in experimental testing: CCS1477 against solid tumors and metastatic prostate cancer (phase I/IIa studies) and hematological malignancies, and CG1350 against multiple myeloma cell proliferation [150].

It should also be mentioned that some inhibitors target different steps of the HIF pathway. For example, PX-478 acts at three levels: decreases HIF-1α transcription, translation, and inhibits de-ubiquitination, favoring protein degradation [151]. Similarly, bortezomib, a proteasome inhibitor, acts as a repressor of HIF-1α transcription, translation, and blocks the recruitment of the coactivator p300 [152,153]. Multiple myeloma is treated with this drug [154]. The Ca^2+^ channel blocker NNC 55-0396 represses HIF-1 activation, increases HIF-1α hydroxylation and degradation, and inhibits its de novo synthesis [155]. CRLX-101 represses HIF-1α stability and translation [156]. Glyceollins block HIF-1α translation and stability via different mechanisms, inhibition of the Pi3k/AKT/mTOR pathway and decreasing HSP90 binding [157].

In addition to the HIF pathway steps previously reported, other components of the HIF system that mediate the hypoxic response mechanism may be potential drug targets. For example, drugs that promote PHD activity or strengthen the interaction between PHD and HIF, and pharmacological inhibition of mitochondrial respiration.

Another strategy to achieve some of the previous interactions is the use of peptides based on the amino acids sequence of HIF-α isoforms. In fact, once introduced into the cell, a competition between peptides and endogenous HIF-α is established, resulting in inhibition of HIF activity or their association with inhibitory proteins. Knowledge of HIF-α structural domains (DNA/binding and transactivation/regulatory) is very useful to model such peptides. Transduction technology is usually used to deliver peptides inside cells. Different examples of peptides that inhibit HIF heterodimerization have been reported. Polypeptides corresponding to the bHLH, PAS, and N/TAD, or the bHLH and PAS of HIF-3α domains of different length form inactive complexes that inhibit interaction with HRE of target genes [158,159]. An HIF-1α-derived peptide reduces binding of endogenous HIF to the target genes, resulting in reduction of HIF-1α activity. Pancreatic cancer cells and xenograft animal models treated with this variant exhibit a reduction of glucose uptake and cell growth [160]. TAT-Ainp1 peptide, hampering the bHLH domain of ARNT, suppresses HIF-1 activity [161]. The cyclic hexapeptide-CLLFVY fused with TAT epitope shows a reduction of HIF-1α/ARNT association and decreased HIF-1 activity [162]. Other peptides have been investigated in the context of HIF-dependent transactivation. Peptides from phage display libraries and synthetic HIF-1α were investigated for their ability for binding to p300. These studies allowed to identify and develop some peptidomimetic HIF-1α/p300 inhibitors [163,164]. Since phosphorylation of the ERK-targeted domain (ETD) in HIF-1α by ERK1/2 is activated in human cancers [165] to allow HIF-1α accumulation into the nucleus, peptides that target this step have also been tested. After initial observation of HIF-1α inhibition by the competitor-peptide ETD, subsequent studies revealed that fusion of ETD with HIV-1 trans-activator of transcription (TAT) was able to inhibit the ERK-dependent transcriptional activity of HIF-1α (but not HIF-2α). The TAT/ETD inhibition, evident only under hypoxia, affects some cancer cell properties: metabolic adaptation, migration, and induction of apoptosis. 

Finally, other approaches have also been proposed, including hypoxia-activated prodrugs (HAPs) [166]. HAPs are first enzymatically converted by one-electron reduction into a prodrug radical anion. In well-oxygenated tissues, the reduction event is reversible. Conversely, under hypoxia, the radical anion either fragments or is reduced further to an active cytotoxin that kills the hypoxic cells engaging a pharmacological target. In this way, HAPs exploit the low levels of oxygen to achieve selectivity [167]. The nitroimidazole mustard evofosfamide (TH-302) is the most advanced HAP, and it is in phase III trials for pancreatic adenocarcinoma and soft tissue sarcoma [168,169]. Praziqual (E09) has been recommended in bladder cancer surgery [170].

Taken together, these data show that the modulation of the HIF pathway in cancer therapy is very attractive and promising. Thus, a detailed understanding of each step of the pathway as well as the specific role of each isoform is essential to develop new and effective drugs.

## 6. Conclusions

Many studies have highlighted a complex metabolic reprogramming occurring in cancer cells which often face hypoxia. HIFs are transcriptional complexes acting as primary transducers of oxygen levels through oxygen-sensing PHD enzymes. In a PHD-mediated manner, HIFs control countless cellular functions, including proliferation and metabolism. 

In the present review, we have highlighted the importance of the HIF-1 system in the adaption of cancer cell metabolism to hypoxia, a process that is essential to promote cancer cell survival, proliferation, and metastasis. The impact of hypoxia is particularly evident on mitochondria and mitochondrial metabolism, including changes in ROS production and signaling. In the last two decades, different studies have investigated the role of HIF-1 in the metabolic reprogramming of different pathways, including glycolysis, glycogen synthesis, lipid metabolism, ETC and TCA cycles, glutamine and serine, ROS production, as well as biogenesis and autophagy of mitochondria. The resulting interplay between HIF-1 and mitochondria is crucial to face the hypoxic condition of tumor cells: oxygen homeostasis in hypoxia is ensured since HIF-1 suppresses mitochondrial oxidative metabolism by reducing oxygen consumption.

Thus, significant developments have been made towards understanding the role of HIF-1 in cancer cells as a master regulator of cancer progression and as a potential target for cancer therapy. However, different aspects of HIF members need to be clarified. For example, the interaction of HIF-1α with other members of the family (HIF-2α and HIF-3α) in the adaption process during hypoxia, and the specific role of each member of the family. Understanding the regulatory mechanism is very important for identifying specific therapeutic targets. Strictly connected with HIF, targeting hypoxia is a potential therapy to face the progression of various cancers and allow long-term survival for patients.

## Data Availability

Not applicable.
